# Knockdown of HMGA2 regulates the level of autophagy via interactions between MSI2 and Beclin1 to inhibit NF1-associated malignant peripheral nerve sheath tumour growth

**DOI:** 10.1186/s13046-019-1183-2

**Published:** 2019-05-03

**Authors:** Kang Yang, Wei Guo, Tingting Ren, Yi Huang, Yu Han, Hongliang Zhang, Jie Zhang

**Affiliations:** 1 Musculoskeletal Tumor Center, Peking University People’s Hospital, No.11 Xizhimen South Street, Beijing, 100044 People’s Republic of China; 2 Beijing Key Laboratory of Musculoskeletal Tumor, Beijing, People’s Republic of China; 3 Department of Urology, Sir Run Run Shaw Hospital, Zhejiang University School of Medicine, Hangzhou, People’s Republic of China

**Keywords:** NF1 MPNST, HMGA2, MSI2, Growth, Apoptosis, Autophagy

## Abstract

**Background:**

Malignant peripheral nerve sheath tumours (MPNSTs) are sarcomas of Schwann cell lineage origin that occur sporadically or in association with the inherited syndrome, neurofibromatosis type 1 (NF1). This study aimed to examine the function of High mobility group protein A2 (HMGA2) in NF1 MPNST progression and the underlying molecular mechanism.

**Methods:**

Immunohistochemistry (IHC) was used to detect HMGA2 expression in MPNST and neurofibroma patient samples. Cell Cycle Kit-8 (CCK-8) and 5-ethynyl-20-deoxyuridine (EdU) assays, terminal deoxynucleotidyl transferase-mediated nick end labelling, and transmission electron microscopy were performed to reveal HMGA2 functions in NF1 MPNST cells in vitro and in vivo. Chromatin immunoprecipitation sequencing (ChIP-Seq) and RNA sequencing (RNA-Seq) were used to detect HMGA2-modulated genes regulating autophagy and growth in NF1 MPNSTs in vitro and in vivo.

**Results:**

NF1 MPNST samples exhibit higher HMGA2 positivity rates (13/16) than sporadic MPNST (16/41) and neurofibroma (0/7) patient samples. High HMGA2 expression is correlated with poor prognosis. Neurofibromin 1 (*NF1*)-deficient MPNST cells display elevated HMGA2 expression. Functional experiments revealed that HMGA2 knockdown inhibits NF1 MPNST cell growth in vitro and in vivo. In addition to promoting cell cycle arrest and apoptosis, HMGA2 knockdown inhibits autophagy, favouring cell death. RNA-Seq and ChIP-Seq revealed that HMGA2 directly activates the Musashi-2 (MSI2) promoter region, and MSI2 overexpression reverses autophagy and growth in shHMGA2-transfected cells. MSI2 interacts with Beclin1, and Beclin1 blockade inhibits autophagy, thereby inhibiting cell proliferation.

**Conclusions:**

HMGA2 knockdown regulates autophagy via MSI2-Beclin1 interactions to inhibit NF1 MPNST growth, revealing potential therapeutic targets for these untreatable tumours.

**Electronic supplementary material:**

The online version of this article (10.1186/s13046-019-1183-2) contains supplementary material, which is available to authorized users.

## Introduction

Malignant peripheral nerve sheath tumours (MPNSTs) are aggressive, incurable sarcomas that arise in 0.001% of the general population as two subtypes: neurofibromatosis type 1 (NF1)-associated and sporadic [1]. In NF1 MPNSTs, benign peripheral nerve plexiform and subcutaneous neurofibromas can undergo malignant transformation to MPNSTs [2, 3]. When this malignant transformation to MPNST occurs, patients have a poor prognosis due to the strong metastatic proclivity of these tumours and their resistance to radiation and chemotherapy [4].

NF1 MPNSTs are generally accepted to be derived from the Schwann cell (SC) lineage upon encountering a second hit. As the first hit, cells of the SC lineage lose expression of the NF1 gene, which encodes Neurofibromin1 (*NF1*), a RAS-GAP. Loss of *NF1* delays RAS-GTP hydrolysis [5–7]. As the second hit, *NF1*-null cells of the SC lineage acquire additional mutations in genes encoding proteins such as PTEN, SUZ12, CDKN2A and RB, which leads to malignant transformation [8–10].

The High mobility group protein A (HMGA) family has emerged as a central player in organ growth regulation and tumourigenesis [11–16]. This family includes HMGA1a, HMGA1b and HMGA2. HMGA2 is a structural transcription factor that is involved in gene transcription regulation, chromatin condensation, DNA damage repair and a series of nuclear events affecting cell proliferation, growth, differentiation, ageing and cell death [17–19]. HMGA2 proteins are abundant during embryogenesis and in many malignant neoplasms, such as myeloproliferative neoplasms and pancreatic, thyroid and ovarian cancer [20–23]. However, HMGA2 expression is absent or markedly diminished in adult tissues.

Recent whole-gene expression profiling identified higher HMGA2 expression in MPNSTs than in normal nerves [5], but the function of HMGA2 in MPNST is still unknown. Here, we sought to understand whether the suppression of HMGA2 can inhibit NF1 MPNST progression and to elucidate the molecular mechanisms.

## Materials and methods

### Clinical specimens

Twenty-two fresh tissue samples (including 8 NF1 MPNST, 8 sporadic MPNST and 6 neurofibroma samples) were collected under protocols approved by the Ethics Committee of Peking University People’s Hospital. Sixty-four paraffin-embedded tissue samples (including 16 NF1 MPNST, 41 sporadic MPNST and 7 neurofibroma samples) were acquired from the Department of Pathology, Peking University People’s Hospital (Beijing, China). The clinical characteristics of these 64 paraffin-embedded samples are shown in Table 1. Clinical and histopathologic information was recorded through a retrospective review of patient records.

**Table 1:** Association between clinicopathologic characteristics of HMGA2 and MSI2 expression

Clinicopathological variables			HMGA2 expression		*p*-value		MSI2 expression		*p*-value
		N	Positive	Negative		N	Positive	Negative	
Sex	Male	31	9	22	0.0259	31	14	17	0.6312
	Female	33	19	14		35	18	17	
Age(year)	≥40	33	13	20	0.6147	33	21	12	0.079
	<40	31	15	16		31	12	19	
Pathogenic site	Extremities	30	10	20	0.2114	34	19	15	0.6167
	Trunk	34	17	17		30	14	16	
Histopathological	Neurofibroma	7	0	7	< 0.01*	7	1	6	0.005*
	NF-1 MPNST	16	13	3		16	13	3	
	Sporadic MPNST	41	16	25		41	18	23	

MPNST:Malignant peripheral nerve sheath tumors; *:*p*<0.05

### Human microarray sample analysis

We collected microarray expression profiles of normal human nerves, neurofibromas and MPNSTs from the Gene Expression Omnibus (GEO) public database, and the accession numbers are GSE41747 and GSE66743. Normalized values from these datasets were analysed for gene expression scores. The expression levels of HMGA2 and Musashi-2 (MSI2) in nerves, neurofibromas and MPNSTs were plotted using SPSS 20.0. *P*-values < 0.05 were considered to indicate a statistically significant difference.

### Cell culture and reagents

The human MPNST cell lines sNF96.2 and sNF02.2 were purchased from ATCC (ATCC, Manassas, VA), while ST8814 and STS26T cells were kind gifts from Dr. Yang Jilong (Tianjin Medical University, China) and Dr. Nancy (Cincinnati Children’s Hospital Medical Center, USA). All cells were cultured in Dulbecco’s modified Eagle’s medium (Gibco) supplemented with 10% FBS at 37 °C in a humidified atmosphere with 5% CO2. Neurofibroma samples were obtained from NF1 patients after informed consent. Tumour specimens were obtained from 3 different patients (2 males and 1 female; age 20–47 years). Separation and culture of neurofibroma Schwann cells (NFSCs) were performed according to the protocol by Thorsten Rosenbaum [24].

Rapamycin and 3-methyladenine (3MA) were purchased from Sigma Chemical Co. LBH589 was purchased from Selleckchem. The following antibodies were used in the experiments: anti-HMGA2, anti-cyclin D1, anti-BCL2, anti-Bax, anti-LC3, anti-p62, anti-Beclin1, anti-PARP, anti-PTEN, anti-ATG12, anti-ATG7,anti-acetyl histone 3 (H3) antibody and anti-GAPDH antibodies from Cell Signaling Technology (Beverly, MA, USA); anti-MSI2 antibodies from Abcam (Cambridge, MA, USA) and Novus Biologicals; and anti-NF1 antibodies from Bethyl (Montgomery, TX, USA).

### Transfection

The lentiviral vectors pLKO.1-HMGA2 (shHMGA2), pLKO.1-Beclin1 (shBeclin1), pLKO.1-MSI2 (shMSI2), pLKO.1-Scramble (shScr), pLVX-Puro-HMGA2 (HMGA2), pLVX-Puro-MSI2 (MSI2) and pLVX-Puro-Control (Ctr) were constructed and used for lentivirus production in HEK293T cells. The NF1 MPNST cell lines ST8814 and sNF96.2 were transduced with lentiviral vectors. Stable cells were selected with puromycin (1.5 μg/ml) for 4 weeks. All primers used in this study are listed in Additional file 3: Table S1.

### Cell cycle Kit-8 (CCK-8) assay

Cells were plated in 96-well plates at a density of 5000 cells in 100 μl of medium per well 1 day before the experiment. Cell viability was examined using CCK-8 (Dojindo Laboratories, Kumamoto, Japan) according to the manufacturer’s instructions.

### Western blotting (WB) analysis

Protein samples were prepared using RIPA lysis buffer [25 mmol/l Tris-HCl (pH 7.5), 150 mmol/l NaCl, 1 mmol/l EDTA, 1% Triton X-100] containing a protease inhibitor cocktail tablet (Roche Applied Science). Proteins were separated via SDS-PAGE and transferred to a nitrocellulose membrane. After blocking with Tris-buffered saline containing 5% skim milk and 0.1% Tween-20 for 1 h at room temperature, the membrane was incubated with primary antibody at 4 °C overnight. The next day, the membrane was washed and incubated with goat anti-mouse or goat anti-rabbit secondary antibody (Boster) for 1 h at room temperature, and enhanced chemiluminescence was used to visualize the protein bands in a Bio-Rad ChemiDoc XRS Imaging System.

### Quantitative real-time polymerase chain reaction (qRT-PCR)

Total RNA was extracted using TRIzol (Invitrogen), and reverse transcription was performed using the Advantage RT-for-PCR Kit (Takara Bio) according to the manufacturer’s instructions. For real-time PCR analysis, dsDNA was amplified using the SYBR Green PCR Kit (Takara Bio). The cycling parameters were as follows: 95 °C for 1 min, followed by 45 cycles of 95 °C for 10 s and 55–60 °C for 30 s. A melting curve analysis was then performed. Cycle threshold (Ct) values were measured during the exponential amplification phase, and amplification plots were analysed using CFX96 software (Bio-Rad). Expression levels were normalized to the fold change in corresponding control cells, which was defined as 1.0. All reactions were performed in triplicate.

### Flow cytometry (FCM) experiments

Cells for cell cycle analysis were fixed in 70% ethanol 1 day before the experiment, digested with RNase A and labelled with propidium iodide (PI). Apoptotic cells were analysed with an Annexin V/FITC kit (BD Biosciences, San Jose, CA, USA) according to the manufacturer’s instructions and analysed by FCM after compound treatment.

### Immunohistochemistry (IHC), immunofluorescence and terminal deoxynucleotidyl transferase-mediated nick end labelling (TUNEL)

IHC was performed as previously described [25]. Paraffin sections were incubated with rabbit polyclonal anti-HMGA2, anti-MSI2, anti-LC3, anti-Beclin1 and anti-Ki67 antibodies (1:100 dilutions). Sections stained with non-immune rabbit serum (1:100 dilution) in phosphate-buffered saline (PBS) instead of primary antibody served as negative controls. Cells exhibiting positive staining at the cell membrane and in the cytoplasm and nucleus were counted in at least 10 representative fields (400× magnification). Immunostaining was assessed by two independent pathologists blinded to clinical characteristics and outcomes.

For immunofluorescence, fixed cells were permeabilized with 0.1% Triton X-100 at room temperature for 15 min and then incubated with anti-LC3, anti-HMGA2, anti-S100b, and anti-MSI2 antibodies overnight at 4 °C. The cells were washed three times with PBS with Tween-20 (PBST) and then incubated for 1 h with Cy3-conjugated goat anti-rabbit IgG and anti-mouse IgG at room temperature. The cells were then analysed using confocal microscopy (Leica STED, Germany).

TUNEL assays were also performed on cells. Apoptotic cells were detected using the ApopTag Plus Peroxidase In Situ Apoptosis Detection Kit according to the manufacturer’s instructions. Stained sections were visualized under a fluorescence microscope.

### 5-Ethynyl-20-deoxyuridine (EdU) assay

EdU assays were performed using the EdU Apollo 567 Cell Tracking Kit (RiboBio, China). Treated and control cells (5 × 10 [3]/well) were seeded onto 96-well plates and incubated with EdU (200 μM) for 2 h at 37 °C. The cells were fixed with 4% paraformaldehyde for 20 min, treated with 0.5% Triton X-100 for 10 min, rinsed with PBS three times, and incubated with 100 μl Apollo reagent for 30 min. Nuclei were labelled with Hoechst 33342. The percentage of EdU-positive cells was calculated based on counts in three independent experiments.

### Transmission electron microscopy

After 72 h of shHMGA2 or 3MA treatment, transmission electron microscopy was performed on cells. Briefly, cells were digested with 0.25% trypsin, suspended at a concentration of 5 × 10^6^ per ml, and fixed at 4 °C for 6 h with 1.5% glutaraldehyde. Next, ultrathin sections (100 nm) were prepared, stained with uranyl acetate and lead citrate and examined under a transmission electron microscope (TEM; H-600; Hitachi, Tokyo, Japan).

### Luciferase reporter assay

Plasmids carrying the MSI2 promoter sequence (− 2417 bp/− 2100 bp) (Luc-MSI2) were synthesized. Luciferase activity was detected using the Dual-Luciferase Assay Kit (Promega Madison, WI) according to the manufacturer’s instructions. Briefly, 1 × 10^5^ cells/well were plated in a 24-well plate. After 12–24 h, the cells were co-transfected with 200 ng expression vector plasmids, 200 ng promoter reporter plasmids, and 50 ng pRL-TK plasmids using Lipofectamine 3000 (Invitrogen) according to the manufacturer’s instructions. Eight hours later, the transfected cells were lysed in culture dishes with lysis buffer, and the resulting lysates were centrifuged at maximum speed for 1 min in an Eppendorf microcentrifuge. The relative luciferase activity of the samples was determined using a Modulus TD20/20 Luminometer (Turner Biosystems), and the samples were normalized for transfection efficiency according to Renilla luciferase activity.

### Immunoprecipitation (IP)

An appropriate amount of antibody was added to cell lysates and then incubated at 4 °C overnight. Protein A/G agarose (Vigorous Biotechnology, Beijing, China; P007) was incubated with cell lysates for 1.5 h. The immunoprecipitates were washed three times using a lysis solution followed by elution with an SDS loading buffer. The eluent was subjected WB.

### RNA sequencing (RNA-Seq) and chromatin immunoprecipitation sequencing (ChIP-Seq)

For RNA-Seq, ST8814 cells were transfected with shHMGA2 or shScr. RNA was isolated (Arcturus PicoPure Kit, LifeTech) and treated with DNase (Qiagen). RNA quality was determined using an Agilent 2100 Bioanalyzer. Library preparation was performed using 150 ng high-quality RNA (TruSeq Library Prep Kit, Illumina), and libraries were sequenced on a HiSeq 2000 (Illumina) by RiboBio Co. (China). RNA-Seq data are available in Additional file 4: Table S2. Each data point is presented as the mean, and all experiments were performed in three biological replicates. Heatmaps of gene expression and Kyoto Encyclopaedia of Genes and Genomes (KEGG) analysis were generated using R language, and KEGG data are available in Additional file 5: Table S3.

For ChIP, 1 × 10^7^ ST8814 cells were crosslinked with 1% formaldehyde for 10 min at room temperature. The samples were sheared using a Covaris sonicator until 200–500 bp DNA fragments were obtained. Then, ChIP was performed using the EZ ChIP™ Kit (Upstate NY, USA) according to the manufacturer’s instructions. DNA was analysed for quality, quantity and size using an Agilent 2100 bioanalyzer and digital PCR. For ChIP-Seq, libraries were sequenced on a HiSeq 2000 (Illumina). ChIP-Seq data are available in Additional file 6: Table S4. The sequencing data were mapped to the mm10 genome, and peak calling was performed using Model-based analysis of ChIP-Seq (MACS) version 1.4.2 with default parameters to obtain primary binding regions. To ensure high-quality, reproducible data, we called peaks with enrichment ≥10-fold over input (*P* ≤ 10^− 9^) and compared the peak sets using ENCODE overlap rules. The heatmaps were generated using Heatmap tools provided by Cistrome. Motif discovery was performed using HOMER. ChIP-Seq datasets (MSI2, CAV1, ZIC1) were subsequently visualized using IGV software.

For ChIP-assay, real-time PCR was performed using quantitative SYBR green PCR mix. Relative fold-enrichments were determined by the 2 − ΔCT methods. Samples were normalized to input chromatin. Anti-acetyl histone 3 (H3) antibody was used as the positive control, and normal rabbit IgG was used as the negative control. Primers for ChIP-qPCR analysis are listed in Additional file 3: Table S1.

### Orthotopic mouse model and in vivo luciferase imaging

Female NSG mice (6 to 8 weeks old) were obtained from SPF Biotechnology Co. Ltd. (Beijing, China). Approximately 5 × 10^4^ cells/5 μl (ST8814) were injected through a microsyringe into the sciatic nerve in mouse thighs to generate tumours for tissue transplantation according to the protocol by Brosius et al. [26] After 4 weeks, the mice were intraperitoneally injected with D-luciferin (Caliper Life Sciences) and allowed to move freely for 10 min to promote substrate absorption. After being anaesthetized, the mice were subjected to whole-body live imaging using the IVIS Imaging System (Caliper Life Sciences). The mice were sacrificed immediately thereafter, and protein was extracted from a portion of the tumour tissue. The remaining tissues were fixed in formalin, embedded in paraffin, sectioned, and stained for IHC.

### Statistical analysis

Data represent the mean ± SD. All statistical analyses were conducted using SPSS 20.0 software package. Statistical tests were one-sided or two-sided, and differences between two groups were assessed using Student’s t-tests, while ANOVA was used to compare multiple groups. Overall survival curves were estimated using the Kaplan–Meier method, and differences in survival were evaluated using the log-rank test. *P* < 0.05 was considered to indicate a statistically significant difference.

## Results

### Elevated HMGA2 expression in human MPNSTs and its relationship with patient survival

**Fig. 1:**
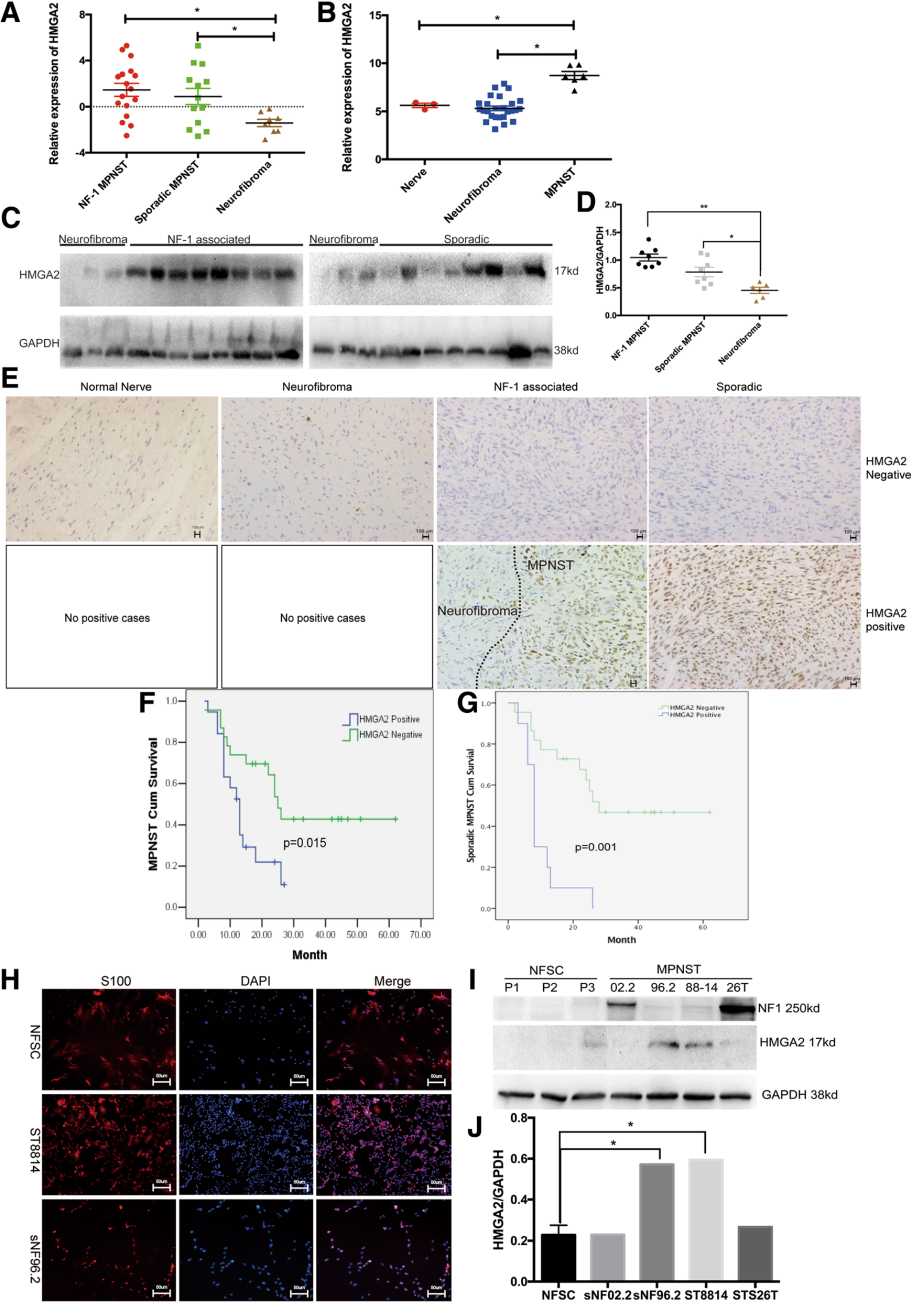
Elevated HMGA2 expression in human MPNSTs and its relationship with patient survival. **a** Average expression of HMGA2 in MPNSTs (*n* = 6) and neurofibromas (*n* = 26) relative to that in normal nerves (*n =* 3) in the Jessen cohort. **b** Average expression of HMGA2 in MPNSTs (*n* = 30) and neurofibromas (*n* = 8) in the Kolberg cohort. **c** HMGA2 protein expression is elevated in MPNSTs compared with that in neurofibromas. **d** GAPDH was used as the control. Relative HMGA2 protein expression is shown as a percentage of GAPDH expression. **e** IHC for positive and negative HMGA2 staining in normal human nerve, neurofibroma, NF1 MPNST and sporadic MPNST sections. Scale bar, 100 μm. **f** and **g** Overall survival of all MPNST patients and sporadic MPNST patients. **h** Identification of human NFSCs by immunostaining for S100b and comparison with the human SC-derived MPNST cell lines ST8814 and sNF96.2. Blue in the merged image indicates DAPI labelling of nuclei. Scale bar, 50 μm. **i** *NF1* protein expression was absent in NFSCs. HMGA2 expression was higher in the *NF1*-deficient MPNST cell lines ST8814 and sNF96.2 than in NFSCs and the *NF1*-expressing cell lines sNF02.2 and STS26T. **j** GAPDH was used as the control. Relative HMGA2 protein expression level is shown a percentage of GAPDH expression. Each data point is presented as the mean ± SD. *P < 0.05. All experiments were performed in three biological replicates

Gene set enrichment analysis (GSEA) of publicly available expression data from two MPNST patient cohorts, Jessen_cohort (GEO: GSE41747) and Kolberg_cohort (GEO: GSE66743) [27, 28], revealed HMGA2 as one of the top significantly enriched oncogenes in MPNSTs compared with that in normal nerves or NF1 neurofibromas (Fig. 1a and b); however, there was no significant difference in HMGA1 expression among nerve, neurofibroma and MPNST tissues (Additional file 1: Figure S1A). We wanted to determine whether HMGA2 is overexpressed in fresh tissue samples. We analysed 6 neurofibroma samples, 8 NF1 MPNST samples and 8 sporadic MPNST samples by WB and found that HMGA2 protein levels were significantly higher in MPNST samples than in neurofibroma samples (Fig. 1c and d). Moreover, using 64 paraffin-embedded tissues, we found that the rate of positive HMGA2 expression was significantly higher in NF1 MPNST samples (13/16) than in sporadic MPNST (16/41) and neurofibroma (0/7) samples (Fig. 1e and Table 1). Next, we examined the correlation between HMGA2 expression and MPNST patient prognosis. Kaplan–Meier survival analysis showed that the overall survival duration of HMGA2-positive patients was significantly shorter than that of HMGA2-negative patients in both the whole MPNST cohort and the subgroup of sporadic MPNST patients (Fig. 1f and g). We also examined the correlation between overall survival and HMGA2 expression in NF1 MPNST patients, since there were only 3 HMGA2-negative patients, and the *P*-value was > 0.05 (Additional file 1: Figure S1B). Then, we generated 3 human NFSC lines, identified by S100b expression (Fig. 1h), and found that *NF1* protein expression was absent in these lines. Furthermore, HMGA2 expression was higher in the *NF1*-deficient MPNST cell lines ST8814 and sNF96.2 than in NFSCs and the *NF1-*expressing cell lines sNF02.2 and STS26T (Fig. 1i and j). We used sNF96.2 and ST8814 as our target cells, because although sNF02.2 was also derived from an NF1 MPNST patient, it expressed full-length *NF1* proteins, and classic NF1 MPNST patients lack *NF1* protein expression.

### HMGA2 knockdown directly leads to the inhibition of NF1 MPNST cell growth via G0/G1 arrest and apoptosis

**Fig. 2:**
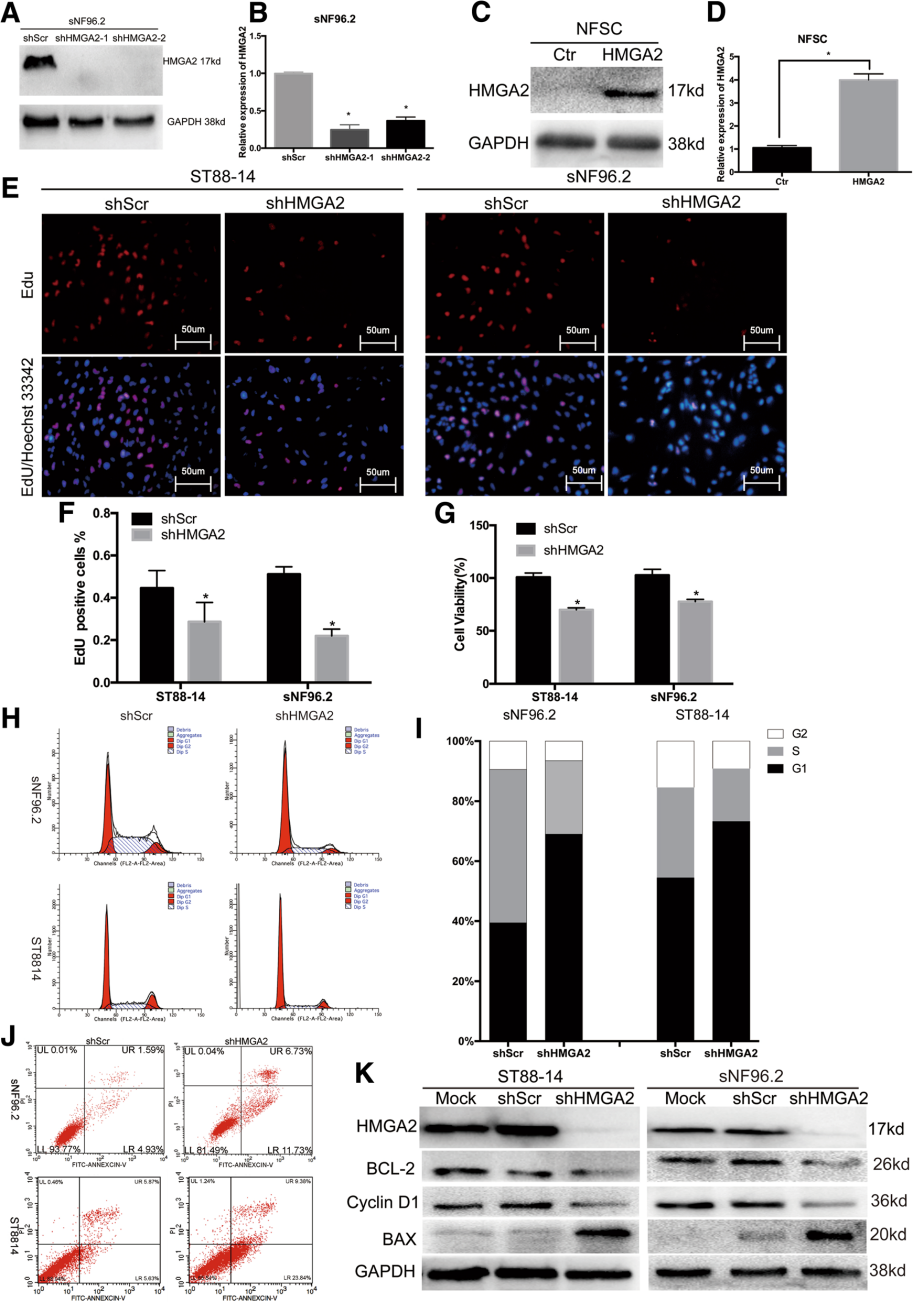
HMGA2 knockdown directly leads to the inhibition of human NF1 MPNST cell growth via G0/G1 arrest and apoptosis. **a** and **b** Two shHMGA2 sequences were used to knock down HMGA2 expression in sNF96.2 cells. Both protein and mRNA HMGA2 expression levels were significantly decreased upon transfection with shHMGA2. **c** and **d** HMGA2-encoding sequences were used to overexpress HMGA2 in NFSCs. HMGA2 expression was significantly increased at both the protein and mRNA levels upon transfection with HMGA2 expression constructs. **e** EdU (red) assays for proliferation rates. Nuclei are stained with Hoechst 33342 (blue). Scale bar = 50 μm. **f** Graphical representation of the proportions of EdU-positive sNF96.2 and ST8814 cells transfected with shScr or shHMGA2. shHMGA2 shows fewer EdU positive cells, indicating that shHMGA2 inhibits cell growth. **g** Cell viability evaluated by the CCK-8 assay. shHMGA2 cells show lower cell viability compared to shScr cells. **h** and **i** Cell cycle analysis performed using FCM. More shHMGA2 cells are in G0/G1 stage compared to shScr cells. **j** Percentage of apoptotic cells determined by FCM. shHMGA2 induces apoptosis more than shScr. **k** Effects of HMGA2 knockdown on G0/G1 phase- and apoptosis-related proteins, as assayed by WB. Each data point is presented as the mean ± SD. *P < 0.05. All experiments were performed in three biological replicates

To determine whether HMGA2 is essential for NF1 MPNST cell growth, we transfected cells with lentiviral vectors encoding HMGA2-targeting shRNAs (shHMGA2) or scrambled control (shScr) and verified the knockdown efficiency (Fig. 2a and b). Decreased cell viability was observed by CCK-8 and EdU assays (Fig. 2e-g). We also transfected HMGA2-overexpressing lentiviral constructs into NFSCs (Fig. 2c and d), but it did not induce NFSC growth (Additional file 1: Figure S1J). EdU labels cells in the S phase, and changes in S phase cells indicate that the cell cycle is also altered. Therefore, cell cycle assays were carried out and revealed that the cells were mostly arrested in G0/G1 phase, implying a reduction in the number of dividing tumour cells following HMGA2 knockdown (Fig. 2h and i). We also detected cell apoptosis by FCM and observed substantial apoptosis in the two cell lines (Fig. 2j).

In addition, the level of the Bax protein, a key executor of cell apoptosis, was increased in NF1 MPNST cells transfected with shHMGA2, as analysed by WB. In contrast, the levels of Bcl2 and the G0/G1 phase-related protein Cyclin D1 were decreased (Fig. 2k).

Altogether, these data demonstrate that HMGA2 is vital for NF1 MPNST cell survival and that repression of HMGA2 leads to tumour cell apoptosis.

### HMGA2 knockdown-induced inhibition of autophagy indirectly promotes NF1 MPNST cell apoptosis

**Fig. 3:**
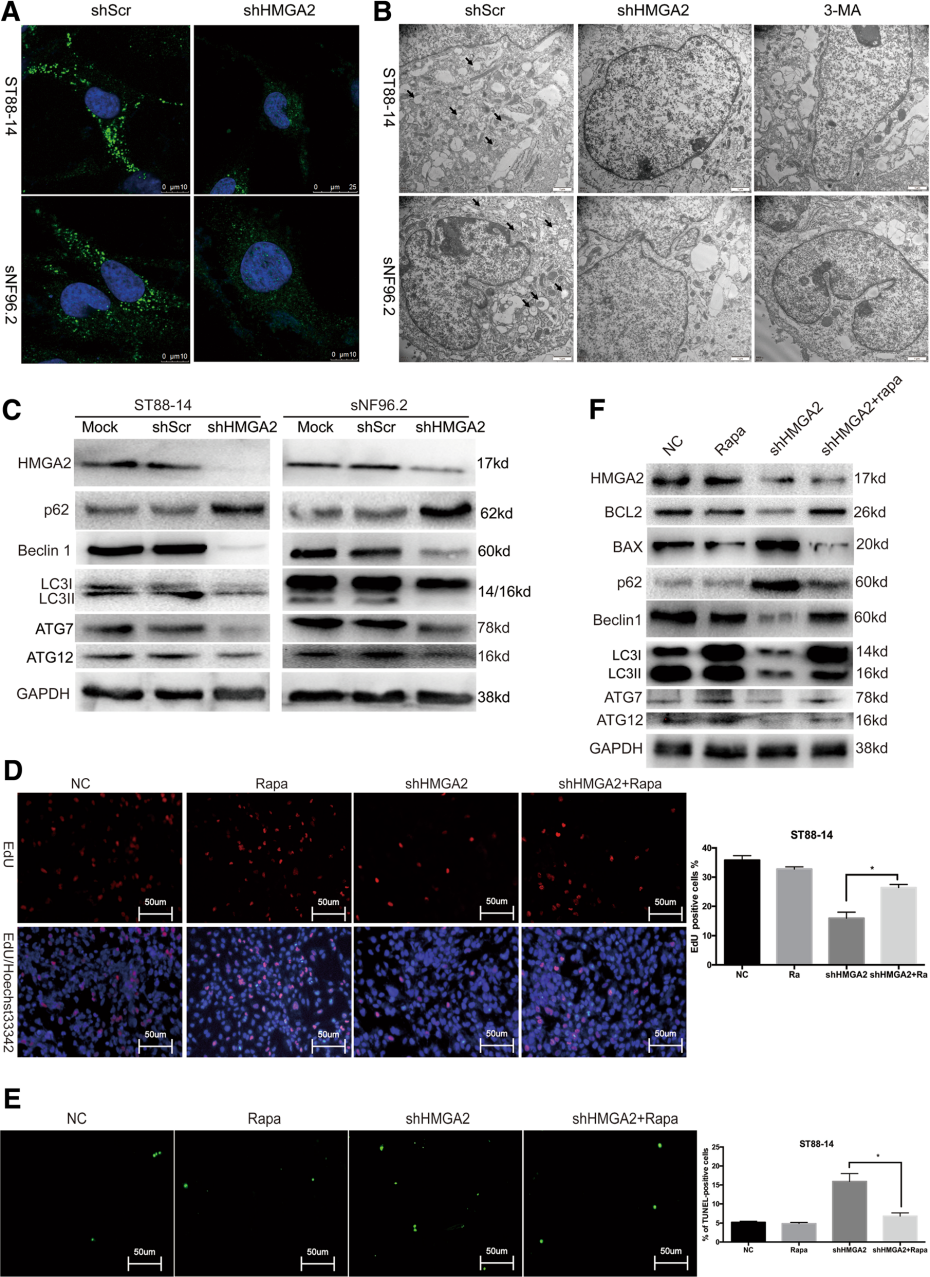
HMGA2 knockdown-induced inhibition of autophagy indirectly promotes NF1 MPNST cell apoptosis (**a**) Cells transfected with shHMGA2 exhibited a punctate pattern of LC3-II fluorescence, with reduced LC3-II expression compared with that in autophagosomes. **b** Representative transmission electron microscopy images depicting the ultrastructures present during autophagy in sNF96.2 and ST8814 cells transfected with shHMGA2 or shScr for 48 h. The images show autophagic vacuoles (arrows) in control cells. No or few autophagic vacuoles were observed in cells transfected with shHMGA2 or treated with 3MA. **c** WB analysis was used to evaluate the expression levels of LC3-II, p62 ATG7, ATG12 and Beclin1. **d** EdU assay revealed that the treatment of cells with rapamycin increased the number of viable HMGA2 knockdown cells. **e** TUNEL positivity of HMGA2 knockdown cells was markedly decreased in the presence of rapamycin. Scale bar = 50 μm. **f** Treatment with rapamycin markedly increased LC3-II, ATG7, ATG12, Beclin1 and BCL2 levels and decreased Bax and p62 levels. Data are presented as the mean ± SD. (n = 3). *P < 0.05 by Student’s t-tests

Autophagy is another form of programmed cell death. To investigate whether HMGA2 is involved in autophagy, we performed TEM analysis to observe cellular ultrastructures present during autophagy. NF1 MPNST cells transfected with shHMGA2 or treated with 3MA exhibited few autophagic vacuoles, whereas a distinct double membrane was present in control cells (Fig. 3b). LC3 is a specific marker of autophagy initiation and is processed from LC3-I to LC3-II during autophagy. Therefore, LC3-II expression can be used to track autophagosome formation by immunofluorescence and confocal microscopy. As shown in Fig. 3a, cells transfected with shHMGA2 exhibited fewer LC3-II fluorescent puncta than did control cells, indicating that autophagy was inhibited by HMGA2 knockdown. Decreased LC3-II, ATG7, ATG12 and Beclin1 expression, accompanied by increased SQSTM1/p62 expression, was clearly detected by WB (Fig. 3c).

Whether autophagy induces or inhibits tumour cell growth depends on the cellular microenvironment [29, 30]. To investigate whether autophagy induces or inhibits the growth of NF1 MPNST cells, we used the autophagy inducer rapamycin, an inhibitor of mTOR. Treatment of cells with rapamycin increased the number of viable HMGA2 knockdown cells, as indicated by EdU assays (Fig. 3d). We also detected apoptotic cells by TUNEL assays and found that treatment with rapamycin markedly reduced the number of HMGA2 knockdown cells (Fig. 3e). Finally, treatment with rapamycin induced LC3-II, ATG7, ATG12 and Bcl2 expression and reduced the levels of SQSTM1/p62 and Bax (Fig. 3f), indicating that autophagy can protect tumour cells from the negative effects of HMGA2 knockdown.

### HMGA2 regulates MSI2 expression in NF1 MPNSTs

**Fig. 4:**
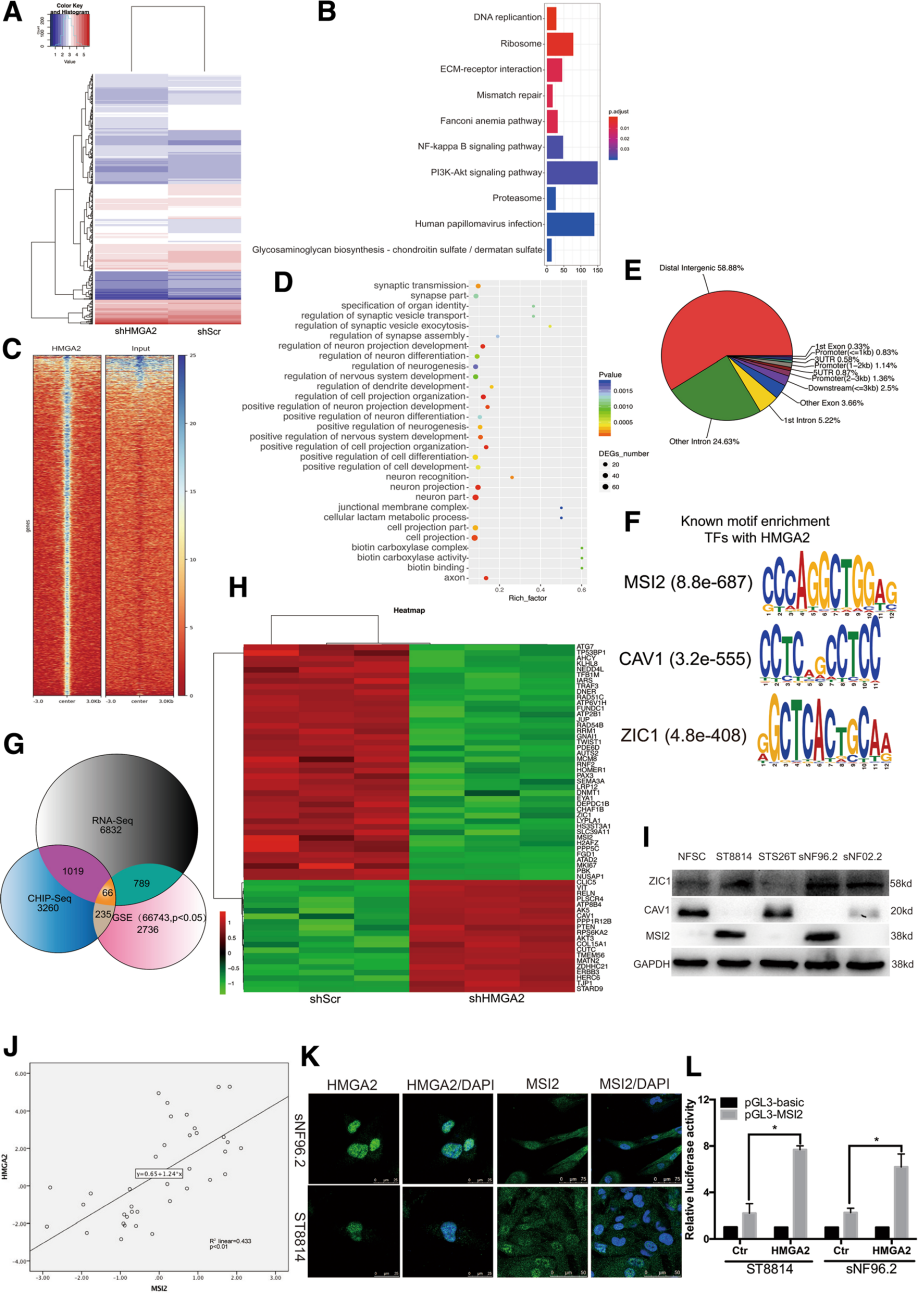
HMGA2 regulates MSI2 expression in NF1 MPNSTs (**a**) Heatmap showing differentially expressed genes in shHMGA2-transfected cells compared with genes in control cells. **b** KEGG analysis of HMGA2-regulated signalling pathways. **c** ChIP-Seq density heatmaps for HMGA2 within ±3 kb of the HMGA2 peak. **d** Gene ontology (GO) analysis of signalling pathways involving genes with potential HMGA2 binding. **e** Pie charts showing the distribution of HMGA2-binding sites in the genome. **f** Motif enrichment of the subgroup with shared increases in chromatin accessible regions. **g** Overlap of genes identified by ChIP-Seq and RNA-Seq and significantly differentially expressed genes in GSE66743. **h** Heatmap showing categories of differentially expressed genes targeted by HMGA2 in NF1 MPNST cells. **i** Verification of the expression levels of genes involved in neural development in five cell lines. **j** Correlation analysis between HMGA2 and MSI2 expression levels in GSE66743. **k** Immunofluorescence analysis of the co-localization and expression of MSI2 and HMGA2 in NF1 MPNST cells. **l** MSI2 promoter-containing luciferase reporter constructs (nucleotides − 2417 to − 2100) were transiently transfected into the indicated cells, and luciferase activity was analysed after 48 h. Data represent the mean ± SD of 3 separate determinations. *P < 0.05 by Student’s t-tests

We next sought to define the mechanisms underlying the role of HMGA2 in NF1 MPNSTs. We transfected ST8814 cells with shHMGA2 and shScr lentiviruses and then performed RNA-Seq profiling (Fig. 4a). We observed inactivation of tumourigenic pathways, including gene sets associated with EGFR, TGF-β, cell cycle, and PI3K-AKT signalling, in the shHMGA2-transfected group (Fig. 4b and Additional file 5: Table S3). We also found that expression of the autophagy gene p62 and the ATG family, such as ATG7, ATG12, and ATG16L1, was altered (Additional file 4: Table S2) and that the autophagy pathway, indicated by KEGG analysis, was inhibited (Additional file 5: Table S3).

To identify the gene targets of HMGA2, we performed ChIP-Seq to assess the genome-wide occupancy of HMGA2 in NF1 MPNST cells (Fig. 4c-f, Additional file 6: Table S4). Then, we overlapped the genes identified by ChIP-Seq and RNA-Seq and significantly differentially expressed genes in GSE66743 (Fig. 4g). We identified three genes, namely, ZIC1, MSI2, and CAV1 (Fig. 4h and Additional file 1: Figure S1C and D), associated with neurodevelopment and found consistent results with the sequencing results in two NF1 MPNST cell lines (Fig. 4i). We also found that the protein levels of ZIC1 and MSI2 were decreased and that CAV1 levels were increased in shHMGA2-transfected cells (Additional file 1: Figure S1E).

Because HMGA2 can bind DNA at AT-rich sites but lacks transcriptional activity, it can positively or negatively modulate the transcriptional activity of several gene promoters by interacting with multiple partners of the transcriptional machinery and consequently altering chromatin structure [13]. MSI2, an RNA-binding protein, is a central regulator of the translation of cancer stem cell programmes [31] and may be a target gene of HMGA2. First, we identified a positive correlation between HMGA2 expression and MSI2 expression through correlation analysis using GSE66743 data (Fig. 4j). Furthermore, we found that MSI2 colocalized with HMGA2 by confocal microscopy (Fig. 4k). ChIP-Seq data showed that HMGA2 binds to the promoter region (Additional file 1: Figure S1C); therefore, we constructed a luciferase reporter plasmid harbouring the promoter region of MSI2. Finally, we observed significantly higher luciferase activity in the HMGA2 overexpression group than in the control group (Fig. 4l).

Altogether, these data demonstrate that HMGA2 can regulate MSI2 expression in NF1 MPNST cells.

### HMGA2 regulates autophagy through MSI2-Beclin1 interactions

**Fig. 5:**
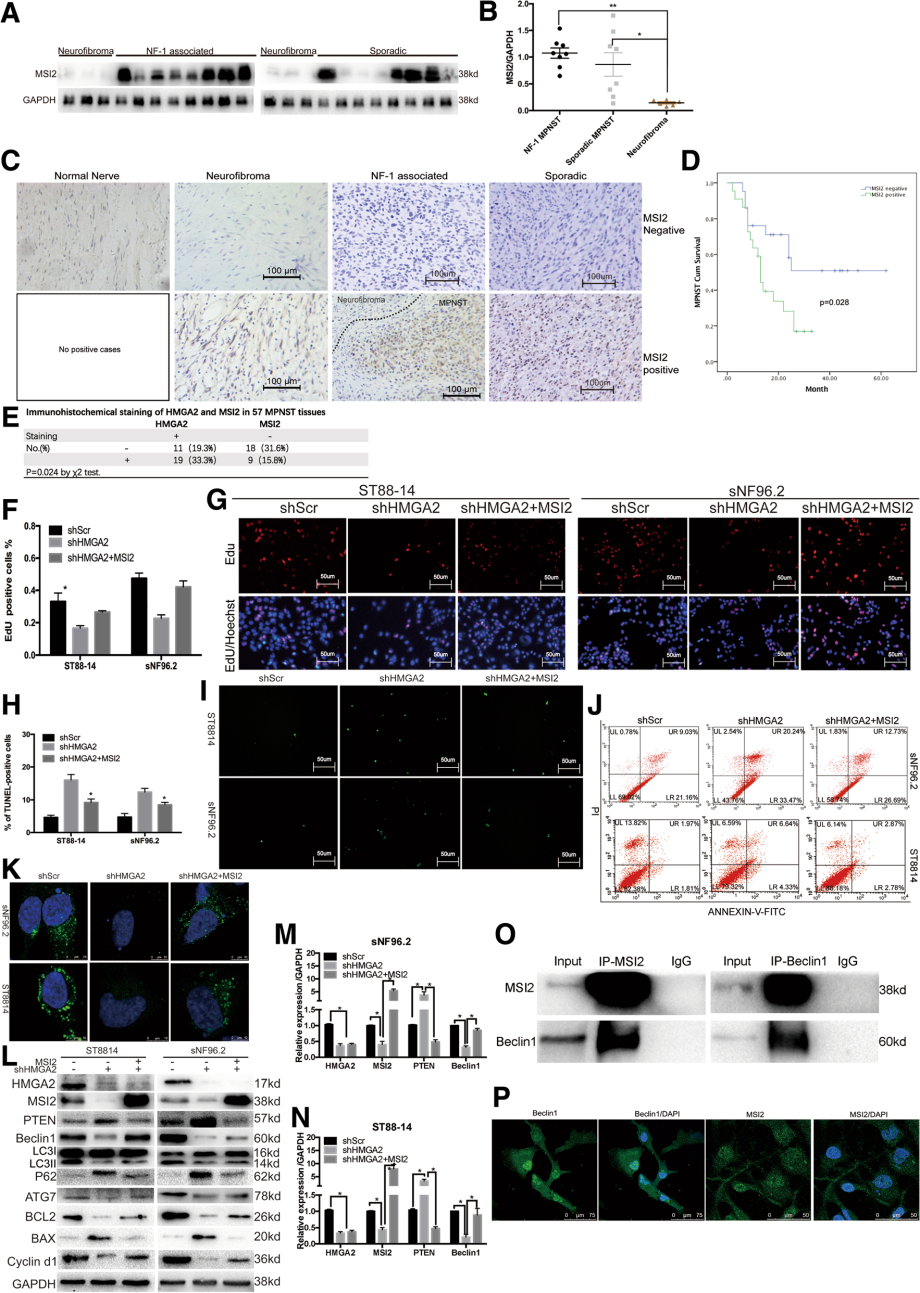
HMGA2 regulates autophagy via interactions between MSI2 and Beclin1 to regulate the growth of NF1 MPNSTs. **a** MSI2 protein expression is elevated in MPNSTs compared with that in neurofibromas. **b** GAPDH was used as the control. Relative MSI2 protein level was expressed as a percentage of GAPDH. **c** IHC for positive and negative MSI2 staining in normal human nerve, neurofibroma, and NF1 MPNST samples compared to sporadic MPNST samples. Scale bar, 50 μm. **d** Overall survival of MPNST patients with positive MSI2 staining. **e** Correlation analysis between positive HMGA2 and MSI2 staining in 57 MPNST tissues. **f** and **g** EdU (red) assays for proliferation rates. Nuclei are stained with Hoechst 33342 (blue). Scale bar = 50 μm. Overexpression of MSI2 reverses proliferation activity of shHMGA2 cells. **h** and **i** TUNEL positivity in HMGA2 knockdown cells was markedly decreased upon MSI2 overexpression. Scale bar = 50 μm. **j** Percentage of apoptotic cells was determined by FCM. Overexpression of MSI2 reverses the apoptosis level of shHMGA2 cells. **k** Cells co-transfected with shHMGA2 and MSI2 showed LC3-II fluorescence puncta with increased LC3-II levels compared to those in cells transfected with shHMGA2 alone. **l** WB was performed to detect the indicated proteins in control-, shHMGA2- and shHMGA2 + MSI2-transfected NF1 MPNST cells. **m** and **n** qRT-PCR analysis was performed to detect HMGA2, MSI2, PTEN, and Beclin1 mRNA expression in control-, shHMGA2- and shHMGA2 + MSI2-transfected NF1 MPNST cells. Bars represent the SEM. **P < 0.05 by Student’s t-tests. **o** IP and WB verifying interactions between MSI2 and Beclin1 in NF1 MPNST cells. **p** Immunofluorescence analysis of the co-localization and expression of MSI2 and Beclin1 in NF1 MPNST cells

We aimed to determine whether HMGA2 regulates tumour growth through MSI2. To this end, we first detected MSI2 expression in fresh tissue samples and found a significantly higher expression of MSI2 in MPNSTs than in neurofibromas (Fig. 5a and b). Then, we examined 64 paraffin-embedded tissues by IHC and found that the MSI2 positivity rate in NF1 MPNSTs (13/16) was higher than that in sporadic MPNSTs (18/41) and neurofibromas (1/7) (Fig. 5c and Table 1). We also wanted to examine the correlation between MSI2 expression and MPNST patient prognosis. Kaplan–Meier survival analysis showed that the overall survival of MPNST patients with high MSI2 expression was significantly shorter than that of patients with low MSI2 expression (Fig. 5d). Furthermore, we analysed the correlation between HMGA2 and MSI2 positive staining by IHC and found that the expression of MIS2 and HMGA2 was correlated with each other (Fig. 5e).

Subsequently, we overexpressed MSI2 in HMGA2 knockdown cells and, through EdU assays, found that compared to that in control HMGA2 knockdown cells, proliferation was significantly restored by MSI2 overexpression (Fig. 5f and g). Additionally, TUNEL positivity was markedly decreased in HMGA2 knockdown cells in the presence of MSI2 overexpression (Fig. 5h and i). FCM also revealed a significant reduction in apoptotic cells (Fig. 5j). Increased BCL2 expression was clearly detected by WB, accompanied by decreased Bax levels (Fig. 5l).

We then aimed to ascertain whether MSI2 affects autophagy. We observed autophagosome formation by confocal microscopy, as shown in Fig. 5k. Cells co-transfected with MSI2 and shHMGA2 exhibited more LC3-II fluorescent puncta than cells with transfected with shHMGA2 alone, suggesting that autophagy was induced by MSI2 overexpression. Increased expression of LC3-II, ATG7 and Beclin1 was clearly detected by WB, accompanied by decreased p62 expression (Fig. 5l). We also knocked down MSI2 in two cell lines and found that MSI2 knockdown inhibited autophagy levels (Additional file 1: Figure S1F, G). Thus, we needed to determine how MSI2 regulates autophagy levels and how autophagy regulates cell growth. We used co-immunoprecipitation (co-IP) to identify which autophagy-related proteins interact with MSI2. Using an anti-MSI2 antibody, we enriched for Beclin1, and in turn, using antibodies against Beclin1, we enriched for MSI2 (Fig. 5o). Furthermore, using immunofluorescence, we found that MSI2 co-localizes with Beclin1 (Fig. 5p). Next, we sought to determine how autophagy induced by Beclin1 regulates cell growth. We inhibited Beclin1 using lentiviral vectors, with shScr as a positive control and 3MA as a negative control. Inhibition of Beclin1 could significantly inhibit autophagy and cell growth and promote apoptosis (Additional file 2: Figure S2A, G). Therefore, HMGA2 regulates autophagy through MSI2-Beclin1 interactions.

PTEN is a tumour suppressor gene that is deleted in many tumours, such as colon cancer, lung cancer, breast cancer and MPNSTs [9, 32–34]. MSI2 can bind to the 3′ untranslated region (UTR) of PTEN [35], indicating that PTEN is the target gene of MSI2. Therefore, we evaluated PTEN expression and found that it was increased in HMGA2 knockdown cells compared to that in control cells; however, this increase was abolished in cells co-transfected with MSI2 and shHMGA2 (Fig. 5i-n).

### HMGA2 knockdown results in the growth inhibition of human NF1 MPNST cells via MSI2 inactivation in vivo

**Fig. 6:**
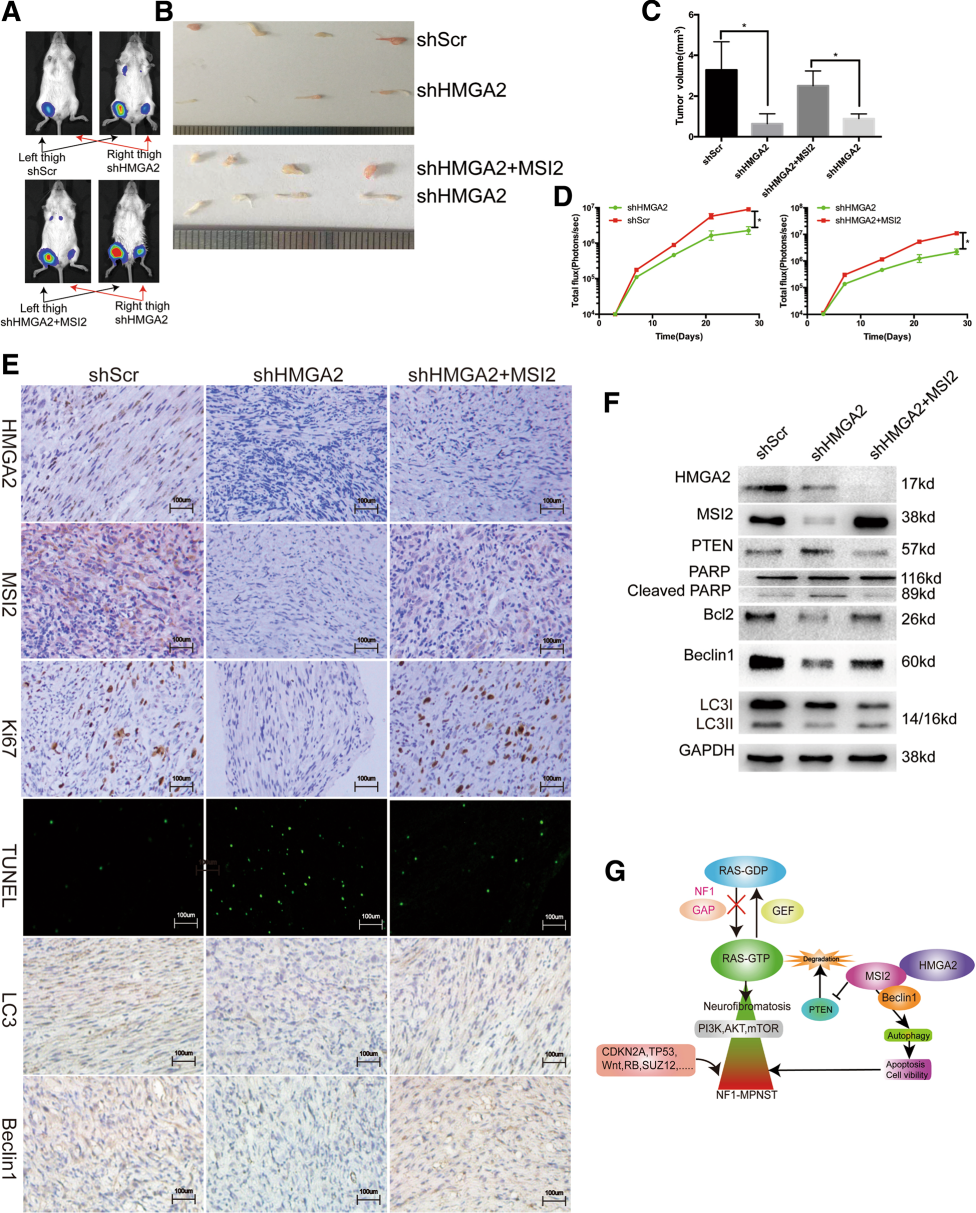
HMGA2 knockdown results in the inhibition of human NF1 MPNST growth via MSI2 signalling inactivation in vivo. **a** Bioluminescence images illustrating a higher concentration of circulating cells in sites injected with control or shHMGA2 and MSI2 co-transfected cells (left thigh) than in sites injected with shHMGA2-transfected cells (right thigh) at 4 weeks. **b** and **c** The stable shHMGA2-transfected group displayed a significant reduction in tumour size. **d** Monitoring of tumour growth. Bioluminescence signals were quantified using Living Image software. The stable shHMGA2-transfected group displayed a significant reduction in tumour growth, while shHMGA2- and MSI2-cotransfected cells exhibited tumour growth reversal. **e** Xenograft tumours were removed, and HMGA2, MSI2, Ki67, Beclin1 and LC3 expression levels were assessed by IHC. Scale bar = 100 μm. The stable shHMGA2-transfected group showed less HMGA2, MSI2, Ki67, Beclin1 and LC3 staining, while shHMGA2 and MSI2 co-transfected cells reverse MSI2, Ki67, Beclin1 and LC3 staining. **f** Xenograft tumours were removed, and the protein expression levels of HMGA2, MSI2, Beclin1, BCL2 and LC3-II were assessed by WB. **g** Schematic representation of HMGA2-MSI2 signalling pathway-induced NF1 MPNST growth

To further verify these in vitro findings, we used an in vivo xenograft model. In the first group (*n* = 4), we injected stable shScr- and shHMGA2-transfected cells into the left and right thighs of the same NSG mice, respectively. In the second group (n = 4), we injected stable shHMGA2 + MSI2- and shHMGA2-transfected cells into the left and right thighs of the same NSG mice, respectively. Compared with the shScr group, the shHMGA2 group displayed significant reductions in tumour size, while these changes were reversed in the shHMGA2 + MSI2 group (Fig. 6a-d). The xenograft tumours were removed and evaluated by IHC, TUNEL and WB. As shown in Fig. 6e, compared with that in the control group, positive staining for MSI2, Beclin1, and LC3 was significantly reduced in the shHMGA2 group, but these changes were reversed in the shHMGA2 + MSI2 group. Moreover, TUNEL positivity was increased in the shHMGA2 group but reduced in the shHMGA2 + MSI2 group. We also validated the conclusions in vitro through WB of xenograft tumours (Fig. 6f).

### LBH589 inhibits cell growth through HMGA2-MSI2 signalling in NF1 MPNSTs

**Fig. 7:**
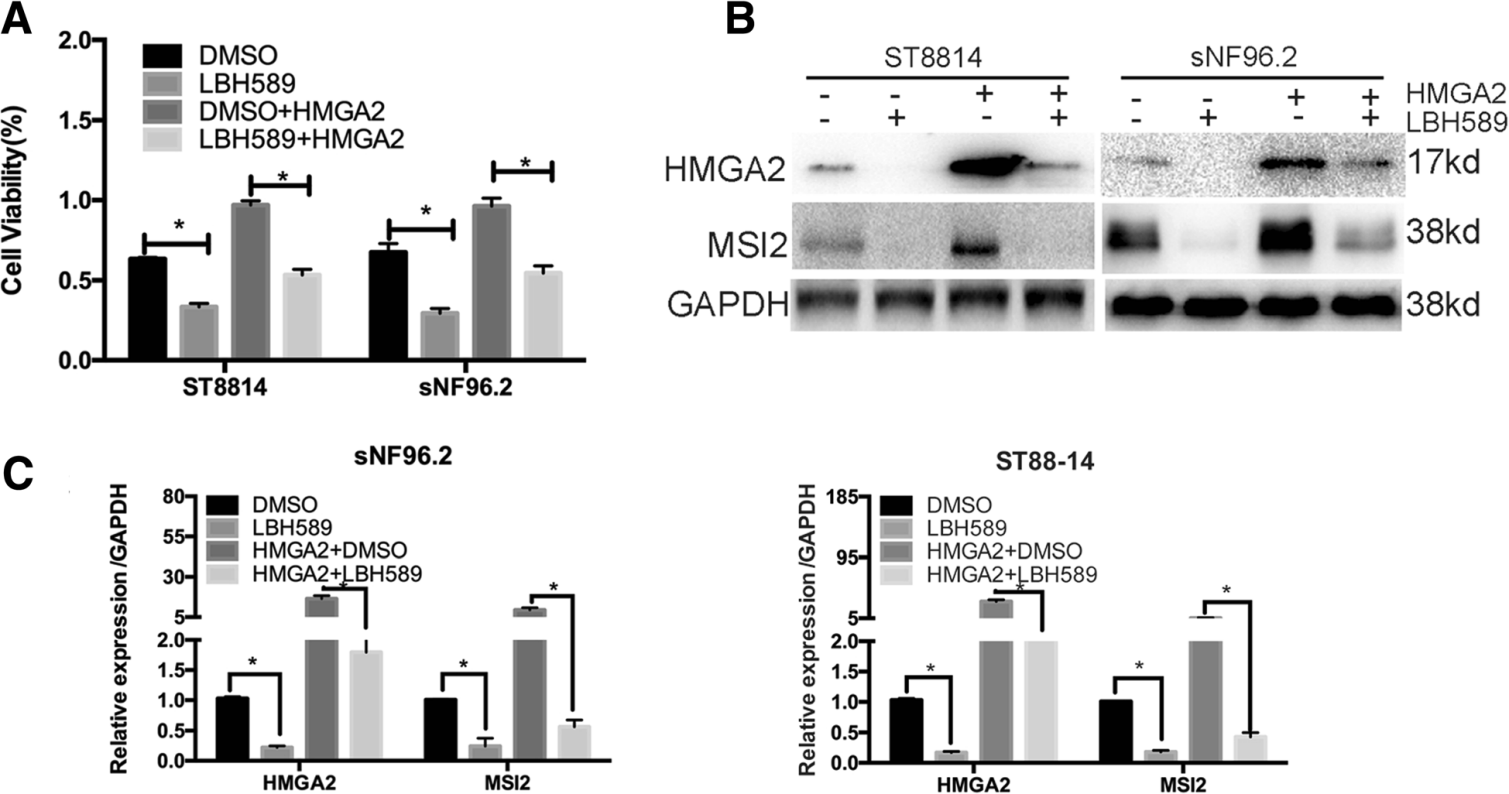
LBH589 inhibits NF1 MPNST cell growth through HMGA2-MSI2 interactions. **a** Cell viability was assessed by CCK-8 assays. LBH589 inhibits cell proliferation activity, while overexpression of HMGA2 reverses cell proliferation activity. The data represent the mean ± SD of three independent experiments. *P < 0.05 by Student’s t-tests. **b** and **c** WB and qRT-PCR were performed to detect the indicated protein and mRNA expression levels in NF1 MPNST cells. Bars represent the SEM. **P < 0.05 by Student’s t-tests

In prostate cancer, LBH589 indirectly inhibits the expression of HMGA2 [36]; thus, we wanted to determine whether LBH589 can also inhibit NF1 MPNST cell growth. We treated ST8814 and sNF96.2 cells with LBH589 and observed decreased HMGA2 expression and decreased cell proliferation by CCK-8 assays (Fig. 7a). However, LBH589 is a histone deacetylase (HDAC)-specific inhibitor and not specific to HMGA2. Therefore, we wanted to determine whether LBH589 regulates cell proliferation through HMGA2. To this end, we overexpressed HMGA2 in cells treated with LBH589 and found that cell proliferation was restored; compared with that in control cells treated with LBH589, where MSI2 levels were decreased, MSI2 expression was increased in LBH589-pre-treated HMGA2-overexpressing cells (Fig. 7b and c), indicating that LBH589 inhibits cell growth through HMGA2 in NF1 MPNSTs.

## Discussion

MPNSTs are highly malignant tumours; some MPNSTs are associated with NF1, and these tumours are mainly caused by the malignant transformation of neurofibromas. First, we confirmed the deletion of SUZ12 in ST8814 cells [10] and found that it was expressed at low levels in sNF96.2 cells and highly expressed in sNF02.2 and STS26T cells. We also examined the effect of overexpressing SUZ12 in ST8814 and sNF96.2 cells and observed no decrease, and even a slight increase, in cell growth (data not shown).

HMGA2 is considered a tumour stem cell marker that is highly expressed in many malignant tumours but not in normal tissues. HMGA2 is highly expressed in MPNSTs and can be used to distinguish this tumour type from synovial sarcomas [5, 37], but the function of HMGA2 in MPNST growth and the underlying molecular mechanism have not yet been studied.

Our analyses revealed that robust HMGA2 activation was more prevalent in human NF1-associated MPNSTs (13/16) than in sporadic MPNSTs (16/41), while HMGA2 was inactive in neurofibromas (0/7). For the first time, we found that HMGA2 knockdown induced G0/G1 cell cycle arrest and apoptosis in NF1 MPNSTs. We overexpressed HMGA2 in NFSCs and found that it did not significantly promote cell growth. The neurofibroma samples we used were from adult patients, and thus, cell growth was very slow, which may explain this finding. Additionally, we evaluated cell growth within 7 days, and as the culture time increases, the cell state deteriorates. However, overexpression of HMGA2 could significantly promote the invasion of NFSCs (data not shown). Autophagy is known to regulate apoptosis [38, 39]. Previous studies have demonstrated that autophagy is common in some malignant tumours and that inhibiting autophagy sensitizes many human apoptosis-resistant tumour cells to chemotherapy [30, 40]. Currently, data describing the effects of HMGA2 on autophagy are scarce, and only one article has reported that HMGA2 can inhibit Cr(VI)-induced autophagy [17]. Here, we demonstrated that HMGA2 knockdown-mediated autophagy inhibition was a cell death mechanism that promoted apoptosis rather than a pro-survival mechanism.

To study the molecular mechanism underlying the role of HMGA2 in NF1 MPNST growth, we used RNA-Seq. We found that HMGA2 knockdown inhibited many tumour signalling pathways, such as the EGFR, TGF-β, cell cycle, and PI3K-AKT signalling pathways. Moreover, we used ChIP-Seq and identified many genes to which HMGA2 may bind, such as TWIST1 and IGF2BP3, which have been reported in previous studies [41]. We also found that HMGA2 binds to autophagy-related genes such as ATG7 and ATG10 (Additional file 6: Table S4). More importantly, by comparing RNA-Seq data, ChIP-Seq data and significantly differentially expressed genes in GSE66743, we found that MSI2, an RNA-binding protein, can be bound and regulated by HMGA2. Previous reports have shown that MSI2 regulates the 3’UTR of HMGA2 in pancreatic adenocarcinoma [42, 43], but based on our results from luciferase reporter assays, the MSI2 promoter region is directly bound by HMGA2, and knockdown or overexpression of MSI2 expression did not affect HMGA2 expression (Fig. 5l and Additional file 1: Figure S1F).

Several studies have indicated MSI2 as a translation regulator that contributes to a variety of cancers [31, 42]. However, little is known about the function and underlying mechanism of MSI2 in MPNSTs. According to our study, MSI2 is highly expressed in NF1 MPNSTs relative to that in neurofibromas. IHC results revealed that MSI2 expression was positively correlated with HMGA2 expression (Fig. 5e). Moreover, concurrent HMGA2 knockdown and MSI2 overexpression confirmed that HMGA2 regulates NF1 MPNST cell growth through MSI2. Further, we found that HMGA2 knockdown inhibits autophagy, whereas MSI2 overexpression in HMGA2 knockdown cells restores autophagy. We also found that MSI2 knockdown inhibits NF1 MPNST cell autophagy and that MSI2 can interact with the autophagy-related gene Beclin1, while inhibiting autophagy by shBeclin1 inhibits cell growth and promotes apoptosis. This study is first to demonstrate that MSI2 interacts with Beclin1 to regulate autophagy and cell growth.

Furthermore, we found that the expression of PTEN, which was shown in a previous report to be regulated by MSI2 via the 3’UTR, was increased upon HMGA2 knockdown, and this increase in expression was suppressed by the concurrent overexpression of MSI2. However, the mechanism by which HMGA2 regulates the growth of NF1 MPNSTs through PTEN requires further study.

Finally, we found that LBH589, an HDAC inhibitor, inhibited the proliferation of NF1 MPNST cells through HMGA2 and may thus be used as a targeted drug to control this tumour.

## Conclusions

Taken together, the results of this study reveal for the first time that knockdown of HMGA2 regulates autophagy levels via interactions between MSI2 and Beclin1 to inhibit the growth of NF1 MPNSTs (Fig. 6g).

## Additional files

Additional file 1: **Figure S1.** (A) Average expression levels of HMGA1 in the Jessen cohort (left). Average expression levels of HMGA1 in the Kolberg cohort (right). (B) Overall survival of NF1 MPNST patients. (C) Binding profiles of HMGA2 on MSI2, CAV1, and ZIC1 loci in ST8814 cells. Shaded areas indicate binding peaks. (D) ChIP assay was performed with a ChIP-grade antibody against HMGA2 to detect the binding of HMGA2 protein to the MSI2, ZIC1, and CAV1 genes in ST8814 cells. Anti-acetyl H3 antibody was used as the positive control, and normal rabbit IgG was used as the negative control. DNA fragments were quantified by qRT-PCR based on input DNA using MSI2-, ZIC1-, and CAV1-specific primers. Each bar represents the mean ± SD from three independent experiments (*P < 0.05 vs. IgG). (E) Knockdown of HMGA2 markedly increased CAV1 protein levels and decreased MSI2 and ZIC1 levels in ST8814 and sNF96.2 cells. (F) Knockdown of MSI2 decreased Beclin1, ATG7, and LC3-II levels and increased p62 levels. (G) Cells transfected with shMSI2 exhibited a punctate pattern of LC3-II fluorescence, with reduced LC3-II expression compared with that in autophagosomes. (H,I) MSI2 labelled with an antibody from Abcam mainly localizes to the nucleus, while that labelled with an antibody from Novus mainly localizes to the cytoplasm. Magnification of the image stained with the anti-MSI2 antibody from Novus reveals that MSI2 is also expressed in the nucleus. (J) Overexpression does not promote NFSC cell proliferation activity. (K):Western blot analysis of HDAC1 E2F1 co-immunoprecipitated with pRB from HMGA2 KD and overexpressed ST8814 cells. Lower panels showing input (lysate) verify HMGA2 knockdown or overexpression efficiency. (L) IHC for HMGA2 and SOX10 staining in NF1 MPNST and sporadic MPNST sections paired to HMGA2 positive staining samples in Fig. 1e. Scale bar, 100 μm. Data are presented as the mean ± SD. (n = 3). *P < 0.05 by Student’s t-tests. (PDF 6618 kb)

Additional file 2: **Figure S2** Knockdown of Beclin1 regulates the growth of NF1 MPNSTs. **Figure S2**A. Two shBeclin1 sequences were used to downregulate Beclin1 expression in sNF96.2 cells. Beclin1 expression at the protein level was significantly decreased upon transfection with shBeclin1. **Figure S2**B. Cells transfected with shBeclin1 exhibited a punctate pattern of LC3-II fluorescence, with reduced LC3-II expression compared with that in autophagosomes. **Figure S2**C and D: EdU (red) assays for proliferation rates. Nuclei are stained with Hoechst 33342 (blue). Scale bar = 50 μm. shBeclin1 inhibited cell proliferation rates. **Figure S1**E and F: TUNEL positivity of shBeclin1 knockdown cells was markedly decreased compared with that of control cells. Scale bar = 50 μm. **Figure S1**G: WB analysis was used to evaluate the expression levels of LC3-II, p62, Beclin1, cleaved-PARP and BCL2. Data are presented as the mean ± SD. (n = 3). *P < 0.05 by Student’s t-tests. (PDF 3617 kb)

Additional file 3: **Table S1.** Primers used in this study. (XLSX 32 kb)

Additional file 4: **Table S2.** RNA-Seq data. (XLSX 2536 kb)

Additional file 5: **Table S3.** KEGG data. (CSV 82 kb)

Additional file 6: **Table S4.** ChIP-Seq data. (XLSX 664 kb)

## Abbreviations

ChIP: Chromatin immunoprecipitation
FCM: Flow cytometry
HMGA2: High mobility group A2
IHC: Immunohistochemistry
IP: Immunoprecipitation
MPNST: Malignant peripheral nerve sheath tumour
MSI2: Musashi-2
NF1: Neurofibromatosis Type 1
*NF1*: Neurofibromin
NFSC: Neurofibroma Schwann cell
p62: SQSTM1/p62
TEM: Transmission electron microscope
TUNEL: Terminal deoxynucleotidyl transferase-mediated nick end labelling
