# Supplementary figures and images for: Knockdown of HMGA2 regulates the level of autophagy via interactions between MSI2 and Beclin1 to inhibit NF1-associated malignant peripheral nerve sheath tumour growth
Source: J Exp Clin Cancer Res. 2019 May 3;38:185. doi: 10.1186/s13046-019-1183-2 (PMC6500071; doi:10.1186/s13046-019-1183-2)

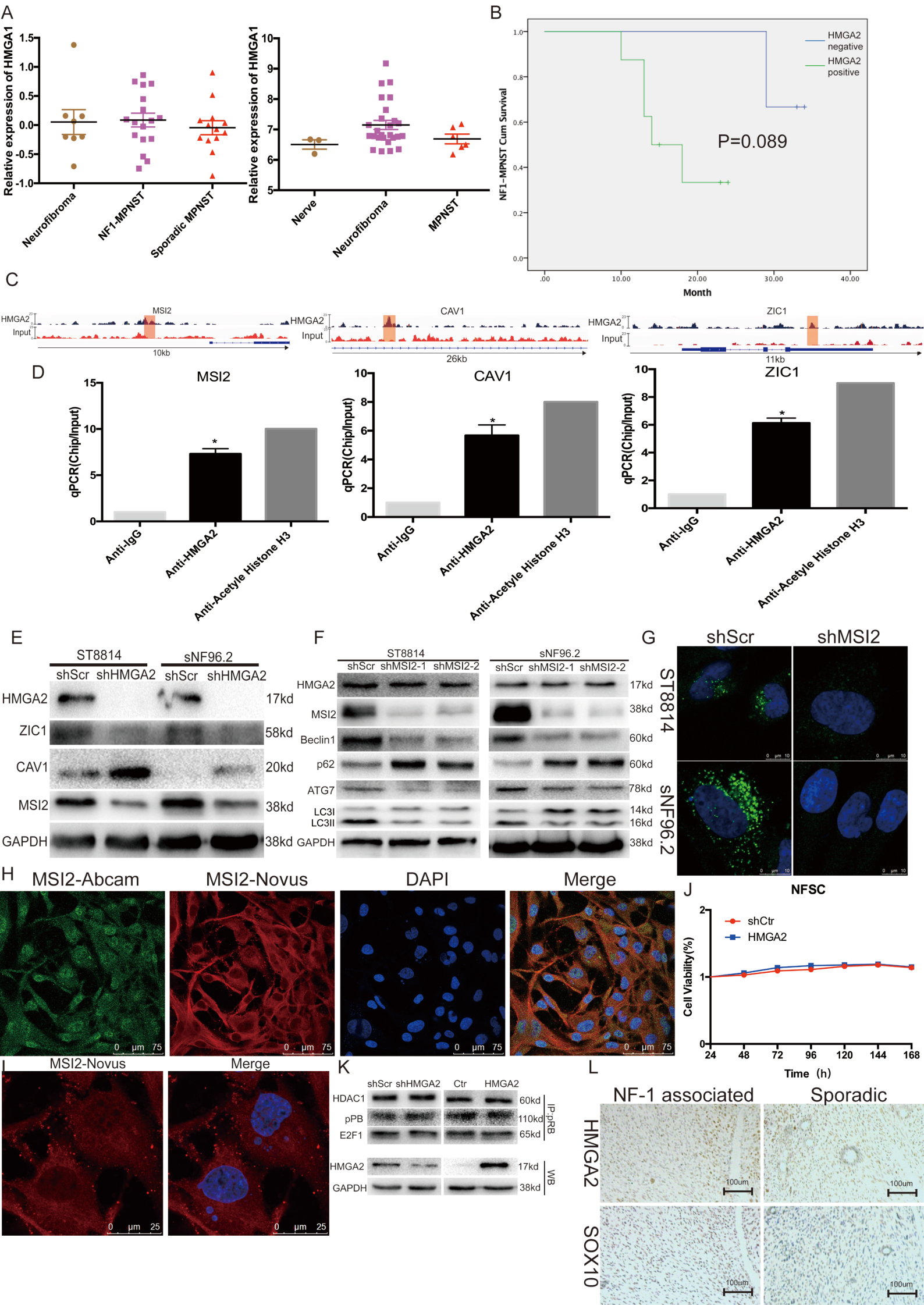

Supplement: Supplementary file 1 — Figure S1. (A) Average expression levels of HMGA1 in the Jessen cohort (left). Average expression levels of HMGA1 in the Kolberg cohort (right). (B) Overall survival of NF1 MPNST patients. (C) Binding profiles of HMGA2 on MSI2, CAV1, and ZIC1 loci in ST8814 cells. Shaded areas indicate binding peaks. (D) ChIP assay was performed with a ChIP-grade antibody against HMGA2 to detect the binding of HMGA2 protein to the MSI2, ZIC1, and CAV1 genes in ST8814 cells. Anti-acetyl H3 antibody was used as the positive control, and normal rabbit IgG was used as the negative control. DNA fragments were quantified by qRT-PCR based on input DNA using MSI2-, ZIC1-, and CAV1-specific primers. Each bar represents the mean ± SD from three independent experiments (*P < 0.05 vs. IgG). (E) Knockdown of HMGA2 markedly increased CAV1 protein levels and decreased MSI2 and ZIC1 levels in ST8814 and sNF96.2 cells. (F) Knockdown of MSI2 decreased Beclin1, ATG7, and LC3-II levels and increased p62 levels. (G) Cells transfected with shMSI2 exhibited a punctate pattern of LC3-II fluorescence, with reduced LC3-II expression compared with that in autophagosomes. (H,I) MSI2 labelled with an antibody from Abcam mainly localizes to the nucleus, while that labelled with an antibody from Novus mainly localizes to the cytoplasm. Magnification of the image stained with the anti-MSI2 antibody from Novus reveals that MSI2 is also expressed in the nucleus. (J) Overexpression does not promote NFSC cell proliferation activity. (K):Western blot analysis of HDAC1 E2F1 co-immunoprecipitated with pRB from HMGA2 KD and overexpressed ST8814 cells. Lower panels showing input (lysate) verify HMGA2 knockdown or overexpression efficiency. (L) IHC for HMGA2 and SOX10 staining in NF1 MPNST and sporadic MPNST sections paired to HMGA2 positive staining samples in Fig. 1e. Scale bar, 100 μm. Data are presented as the mean ± SD. (n = 3). *P < 0.05 by Student’s t-tests. (PDF 6618 kb) [file 13046_2019_1183_MOESM1_ESM.pdf]

A

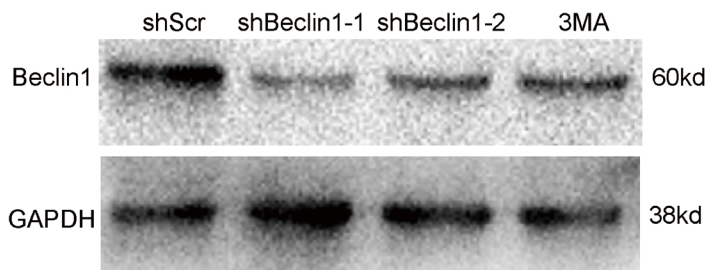

B

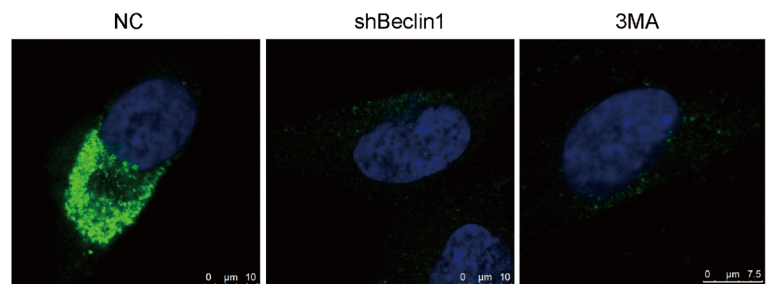

C

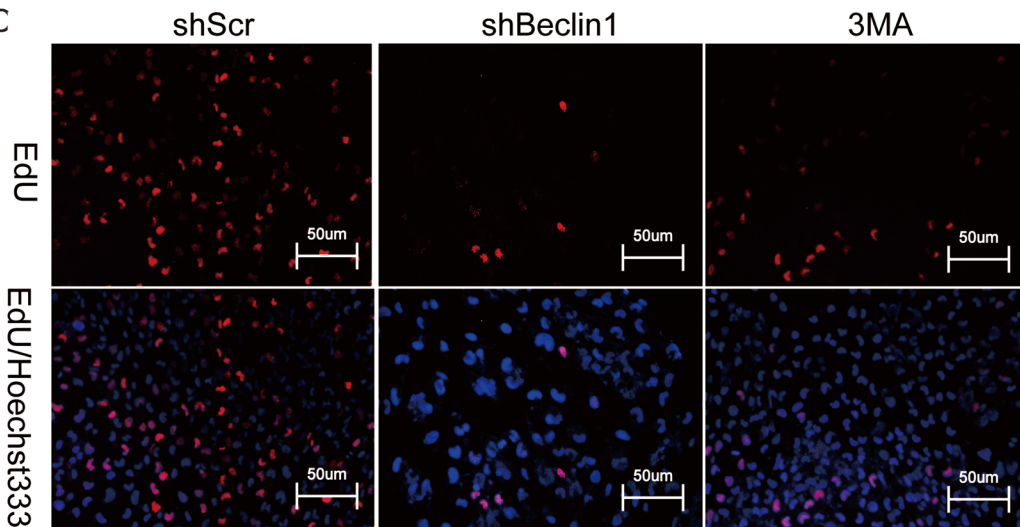

D

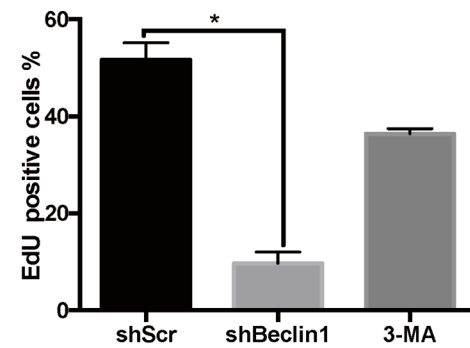

F

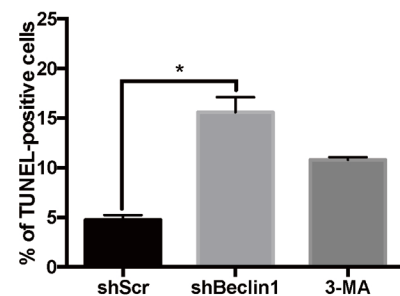

G

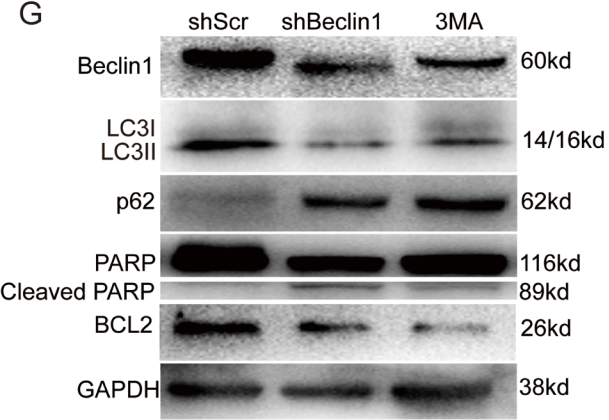

Supplement: Supplementary file 2 — Figure S2 Knockdown of Beclin1 regulates the growth of NF1 MPNSTs. Figure S2A. Two shBeclin1 sequences were used to downregulate Beclin1 expression in sNF96.2 cells. Beclin1 expression at the protein level was significantly decreased upon transfection with shBeclin1. Figure S2B. Cells transfected with shBeclin1 exhibited a punctate pattern of LC3-II fluorescence, with reduced LC3-II expression compared with that in autophagosomes. Figure S2C and D: EdU (red) assays for proliferation rates. Nuclei are stained with Hoechst 33342 (blue). Scale bar = 50 μm. shBeclin1 inhibited cell proliferation rates. Figure S1E and F: TUNEL positivity of shBeclin1 knockdown cells was markedly decreased compared with that of control cells. Scale bar = 50 μm. Figure S1G: WB analysis was used to evaluate the expression levels of LC3-II, p62, Beclin1, cleaved-PARP and BCL2. Data are presented as the mean ± SD. (n = 3). *P < 0.05 by Student’s t-tests. (PDF 3617 kb) [file 13046_2019_1183_MOESM2_ESM.pdf]
